# Visual Recognition of Age Class and Preference for Infantile Features: Implications for Species-Specific vs Universal Cognitive Traits in Primates

**DOI:** 10.1371/journal.pone.0038387

**Published:** 2012-05-31

**Authors:** Anna Sato, Hiroki Koda, Alban Lemasson, Sumiharu Nagumo, Nobuo Masataka

**Affiliations:** 1 Primate Research Institute, Kyoto University, Inuyama, Aichi, Japan; 2 EthoS “Ethologie Animale et Humaine” UMR6552-CNRS, Université de Rennes 1, Station Biologique, Paimpont, France; 3 Institut Universitaire de France, Paris, France; Texas A&M University, United States of America

## Abstract

Despite not knowing the exact age of individuals, humans can estimate their rough age using age-related physical features. Nonhuman primates show some age-related physical features; however, the cognitive traits underlying their recognition of age class have not been revealed. Here, we tested the ability of two species of Old World monkey, Japanese macaques (JM) and Campbell's monkeys (CM), to spontaneously discriminate age classes using visual paired comparison (VPC) tasks based on the two distinct categories of infant and adult images. First, VPCs were conducted in JM subjects using conspecific JM stimuli. When analyzing the side of the first look, JM subjects significantly looked more often at novel images. Based on analyses of total looking durations, JM subjects looked at a novel infant image longer than they looked at a familiar adult image, suggesting the ability to spontaneously discriminate between the two age classes and a preference for infant over adult images. Next, VPCs were tested in CM subjects using heterospecific JM stimuli. CM subjects showed no difference in the side of their first look, but looked at infant JM images longer than they looked at adult images; the fact that CMs were totally naïve to JMs suggested that the attractiveness of infant images transcends species differences. This is the first report of visual age class recognition and a preference for infant over adult images in nonhuman primates. Our results suggest not only species-specific processing for age class recognition but also the evolutionary origins of the instinctive human perception of baby cuteness schema, proposed by the ethologist Konrad Lorenz.

## Introduction

Human physical features (e.g., body size, facial appearance, sexual organs, and hair color) change with age. When interacting with an unfamiliar person, those physical features facilitate the estimation of his/her rough age, or at least age class (e.g., infant, juvenile, young adult, adult, or elder). “Own-age bias” is an example of a cognitive trait used by humans for age recognition. This bias suggests that one's age estimations of an unfamiliar person are more sensitive and precise when they are about one's own age than when they are of a very different age [2]. In humans, identifying the age class of an individual plays an important role in decision making during social interactions and communication; we show respect for elders [3] and tolerate children's mischief [4]. Furthermore, we adapt our way of speaking to very old or very young people [5]. These age-dependent differences in social interactions are a part of high-order social cognitions that have evolved through complex interactions in social environments, particularly in the primate lineage [6], [7], [8].

It is clear that age class plays a key role in nonhuman primate social interactions. Most monkey and ape species form social groups including various age class categories, such as infant, juvenile, adolescent, matured adults, and older individuals, characterized by differences in physical appearance. Humans are able to visually discriminate heterospecific age classes; however, it is unknown whether nonhuman primates are able to visually discriminate conspecific age classes [9]. Most primatologists can readily estimate the age class of animal subjects based on their physical characteristics. During the relatively long social life of nonhuman primates, age class is likely to influence social status in their group. For example, recent findings have shown that elders are privileged interlocutors during vocal communication [10]. Elders also contribute to the community through a stabilizing role [11]. Moreover, infants receive special interest from female group members, an advantage for infant survival [12]. These findings strongly suggest that nonhuman primates, like human primates, are able to spontaneously recognize age classes. However, the ability to visually discriminate age class has not been tested in nonhuman primates.

In the present study, we examined the ability of nonhuman primates to spontaneously discriminate between infant and adult images using visual paired comparison tasks (VPC). The VPC paradigm is based on novelty preference, i.e., visual attention is likely captured by a novel stimulus/object. The paradigm is commonly used in studies on human infants and nonhuman primates to test the ability to discriminate between two comparable stimulus categories [13], [14]. In this task, participants are first required to look at the center of the monitor (fixation phase). Then a single stimulus from a given category is presented to the participants (familiarization phase). This is followed by the test phase during which two stimuli are simultaneously presented, one novel stimulus from the same category as that seen in the familiar phase (familiar stimulus) and one novel stimulus from a novel category (novel stimulus). It is assumed that if the individual can discriminate between the two stimulus categories, more attention (i.e., rapid attention capture by the novel stimuli at the first look [FL] and longer looking time [LT]) will be directed toward the novel stimulus than toward the familiar stimulus. Although matching-to-sample tasks (MTS) based on operant conditioning provide a direct way to test perceptual or recognition ability, they involve intensive and extensive training, particularly for social-cognitive categories such as age class. VPC does not require training and is rapidly applicable to naturalistic stimuli; thus, it is commonly used in the study of visual recognition in human infants [13], [14], [15] and nonhuman primates [16], [17], [18], [19]. A further advantage of VPC is that it allows the comparison of visual attractiveness or preference between paired stimulus categories. If one of the two categories (category A) is more attractive for participants than the other (category B), LTs would be longer when category A is the novel stimulus than when category B is the novel stimulus in the test phase. This asymmetrical effect of familiarization order has been reported in VPC as well as in serial habituation-dishabituation paradigm testing the gender discrimination in both human infants [20], [21], [22] and monkeys [23], [24], [25]. They concluded that the asymmetry of novelty attractiveness is generated by a preference for one of the two stimulus categories. Based on that, we considered the differences in LTs for the infant and adult novel stimuli to indicate visual preference in the novel stimulus category.

The aims of the present study were to examine the ability of nonhuman primates to discriminate between infant and adult images and to investigate the monkeys' preferences for infantile physical features. For humans, infantile features are innately perceived as cute [26], [27], [28], [29], [30], [31]. The ethologist Konrad Lorenz [1] proposed that these infantile features, known as the baby schema (“Kindchenschema”), motivate caretaking behavior. He hypothesized that the human attraction to infantile features is not restricted to conspecifics, but can be generalized to heterospecific stimuli, including young animals and comic characters such as “Mickey Mouse” and “Teddy bear” [32], [33]. To test the evolutionary continuity of the human preference for baby schema, we investigated the visual preference of monkeys to images of infants at intra- and inter-specific levels in two nonhuman primate species, Japanese macaques (*Macaca fuscata*; JM) and Campbell's monkeys (*Cercopithecus campbelli;* CM).

## Results

**Figure 1:**
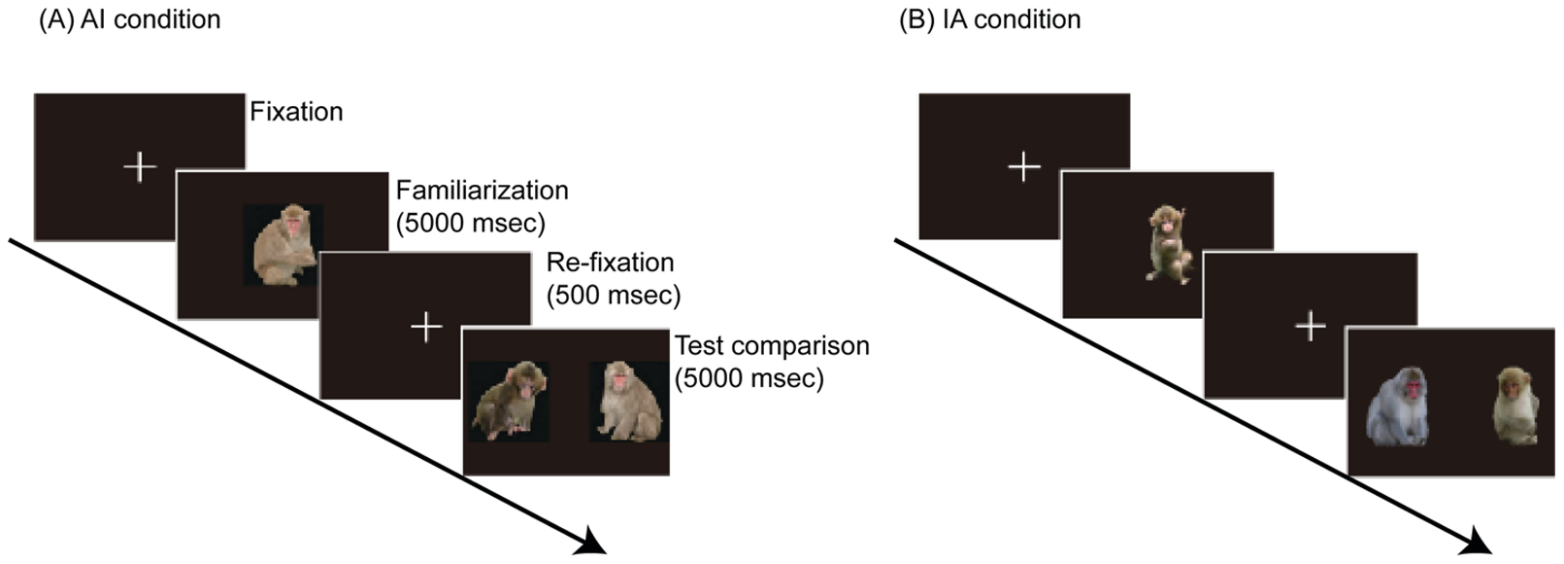
Schematic representation of the VPC paradigm for the (A) adult/infant (AI) condition, and the (B) infant/adult (IA) condition.

In Experiment 1, JMs were used both as subjects and as stimulus images in the VPC tasks. The tasks included two order conditions: adult-infant (AI) and infant-adult (IA). In the AI condition, an adult stimulus was presented in the familiar phase and an infant image was used as the novel stimulus in the test phase; the stimuli were reversed in the IA condition. Figure 1A shows a schematic representation of the fixation, familiarization, re-fixation, and test phases for the AI condition in a single VPC trial.

**Figure 2:**
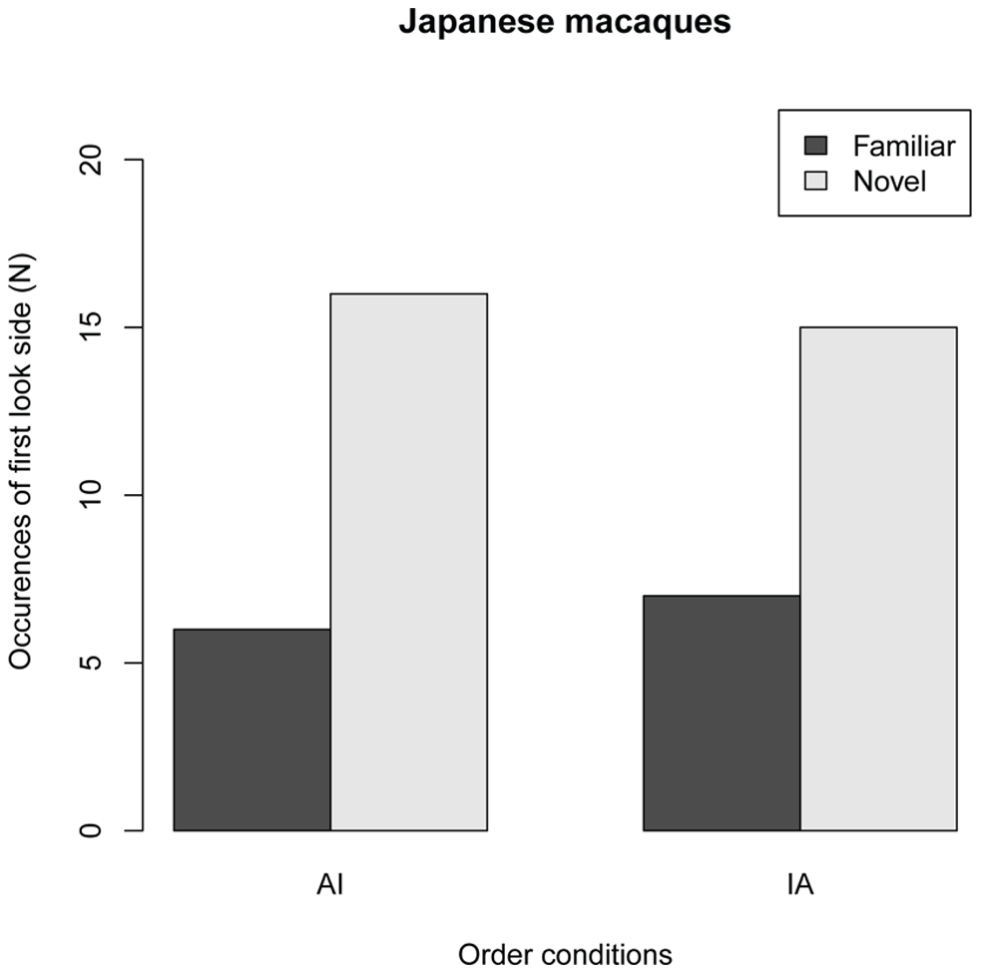
First look sides (FLs) for the stimulus novelty and order conditions in 44 trials of 11 Japanese macaque subjects. In the adult/infant (AI) condition, the adult image was used as the stimulus in the familiar phase and the infant image served as the novel stimulus in the test phase. In the infant/adult (IA) condition, the infant image was used as the stimulus in the familiar phase and the adult imaged served as the novel stimulus in the test phase. Black bars, FLs for familiar stimuli; white bars, FLs for novel stimuli.

**Figure 3:**
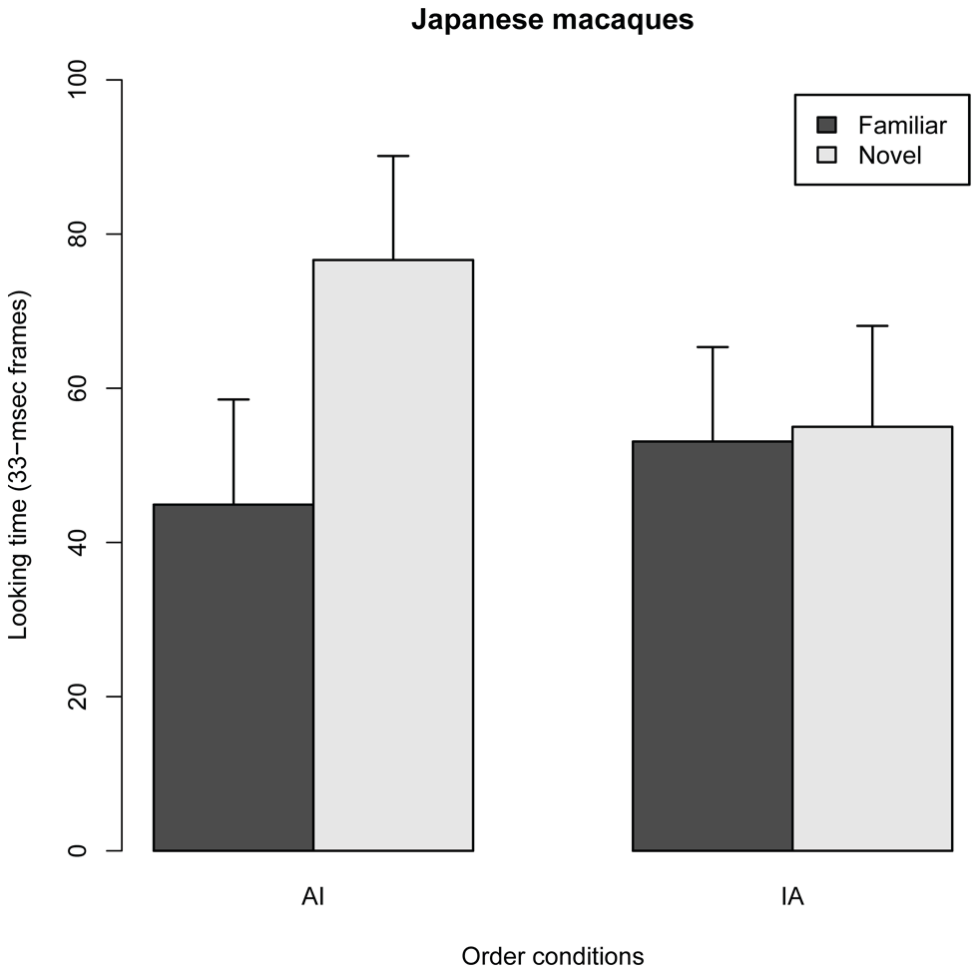
Looking times (LTs) for the stimulus novelty and order conditions in 11 Japanese macaque subjects. In the AI condition, the adult image was used as the stimulus in the familiar phase and the infant image served as the novel stimulus in the test phase. In the IA condition, the infant image was used as the stimulus in the familiar phase and the adult imaged served as the novel stimulus in the test phase. Error bars represent mean values ± 95% confidence intervals. Black bars, familiar stimuli; white bars, novel stimuli.

Figure 2 shows the side of the first look (FL) in the two order conditions in Experiment 1. The GLMM analysis revealed no significant main effect for order condition (Estimated parameter coeffiencet ± se, 0.22±0.66, *z* = 0.33, *P* = 0.74), suggesting that FL patterns were equivalent between AI and IA conditions. Further analysis for intercept of GLMM showed that the probability of FL for novel images were higher than those for familiar ones (Intercept, 0.89±0.33, *z* = 2.63, *P* = 0.0085), suggesting that JM subjects looked first at novel images regardless of order conditions. Figure 3 shows the total look durations (LT) for the novel and the familiar stimuli in the two order conditions in Experiment 1. The GLMM analysis revealed a significant main effect for novelty (F*_1,42_* = 8.16, *P* = 0.0066) but no significant effect for order condition (F*_1,32_* = 1.30, *P* = 0.262); however, an interaction effect was found between novelty category and order condition (F*_1,42_* = 6.41, *P* = 0.0152). This finding suggests that the effect of novelty (LT differences between novel and familiar stimuli) differed between the AI and IA conditions. The analysis of parameter coefficients in the GLMM revealed that the LT for novel images was significantly longer than that for familiar images in the AI condition, whereas no effect of novelty was found in the IA condition (Figure 3, Table 1). This finding indicates that LT for the novel stimulus was significantly longer than that for the familiar stimulus only when the novel stimulus was the infant image. Thus, JMs looked at infant images for a significantly longer time than they looked at adult images. These results demonstrate that JM subjects possess a spontaneous ability to discriminate infant images from adult images. Furthermore, JM subjects preferred looking at infant images over adult images.

**Table 1:** GLMM parameter estimate coefficients in Experiment 1.

Estimated parameter contrasts	Average ± SE	Df	*t-*value	*P*
Difference between novel and familiar images in the AI condition	44.91±8.33	42	3.81	0.0004
Difference between novel and familiar images in the IA condition	1.91±8.33	42	0.229	0.82
Difference between AI and IA conditions for the familiar image	−8.18±8.33	32	0.982	0.33
Difference between AI and IA conditions for the novel image	21.64±8.33	32	2.60	0.014

**Figure 4:**
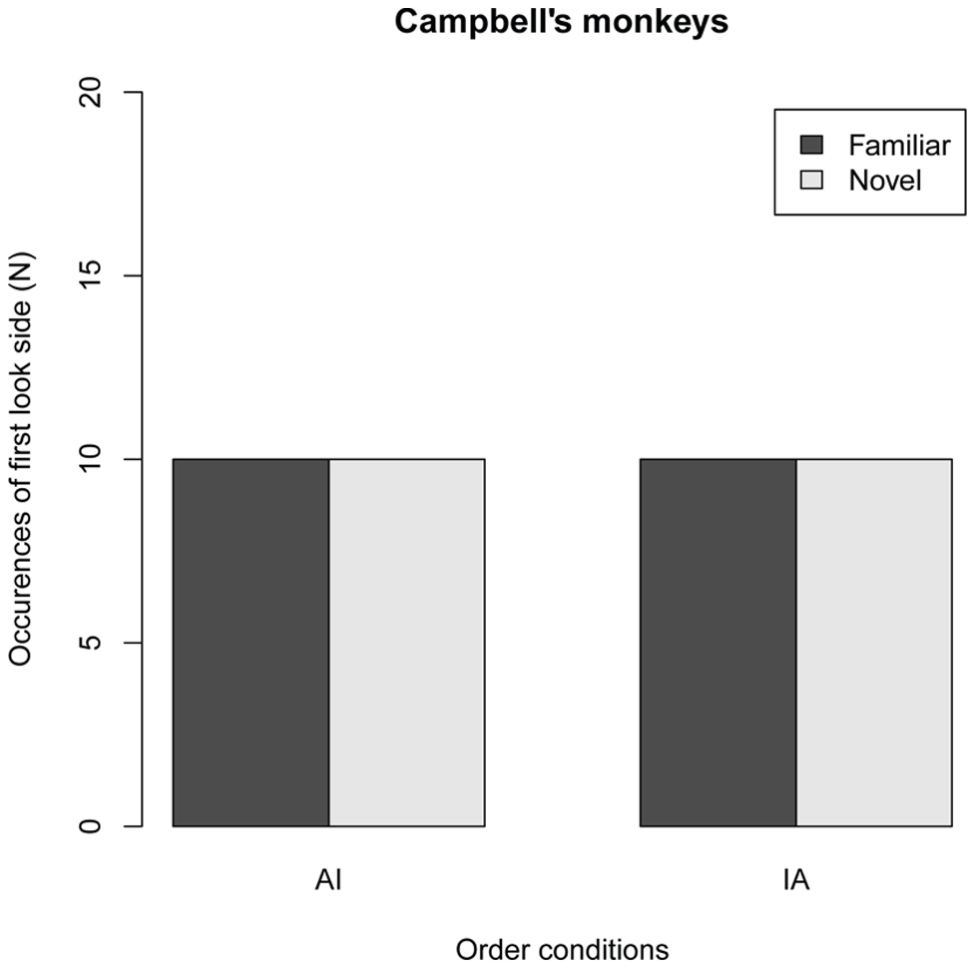
First look sides (FLs) for the stimulus novelty and order conditions in 40 trials of 10 Campbell '**s monkeys.** In the AI condition, the adult image was used as the stimulus in the familiar phase and the infant image served as the novel stimulus in the test phase. In the IA condition, the infant image was used as the stimulus in the familiar phase and the adult imaged served as the novel stimulus in the test phase. Black bars, FLs for familiar stimuli; white bars, FLs for novel stimuli.

**Figure 5:**
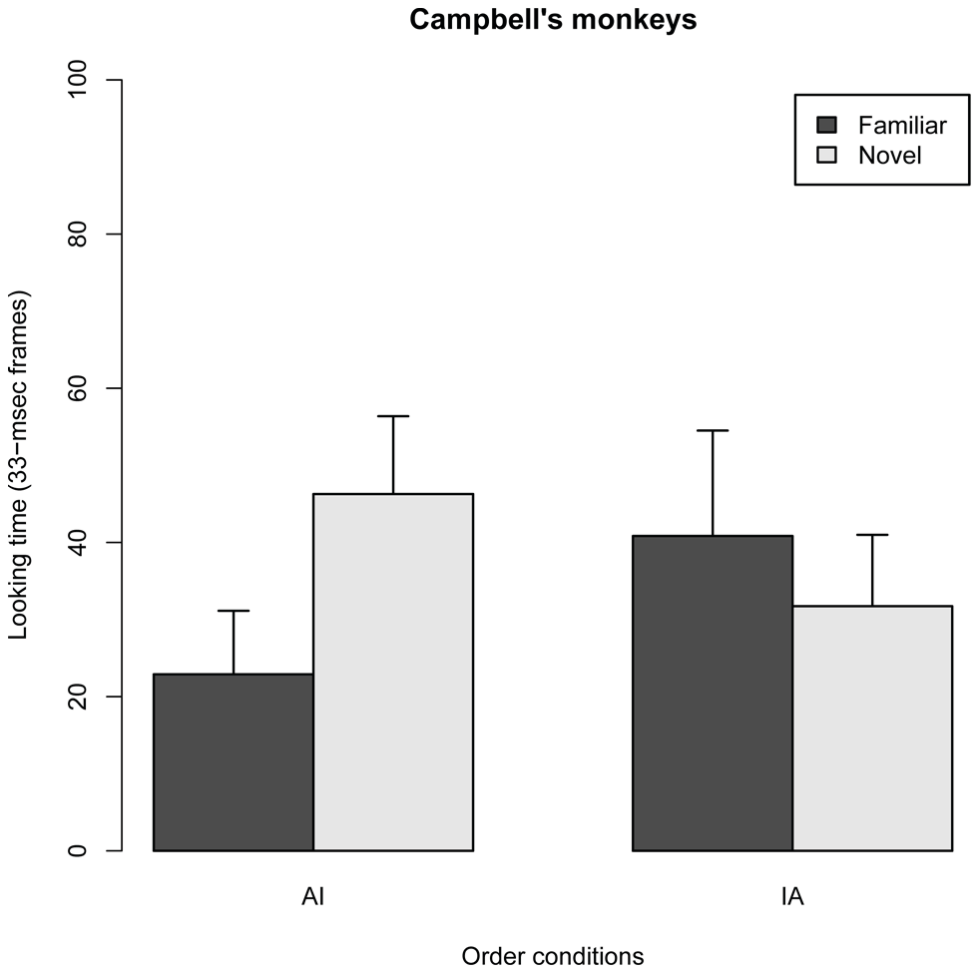
Looking times (LTs) for the novelty stimulus and order conditions in 10 Campbell '**s monkeys.** In the AI condition, the adult image was used as the stimulus in the familiar phase and the infant image was used as the novel stimulus in the test phase. In the IA condition, the infant image was used as the stimulus in the familiar phase and the adult image served as the novel stimulus in the test phase. Error bars represent mean values ± 95% confidence intervals. Black bars, familiar stimuli; white bars, novel stimuli.

In Experiment 2, CMs were presented with the same JM image stimuli in the VPC tasks. Figure 4 shows the side of the first look in the two order conditions in Experiment 2. The occurrences of FLs for novel stimulus were the same with those for familiar stimulus, in both IA and AI conditions, indicating that FLs of CM subjects were not influenced by the stimulus category in the familiarization phase. This FL results would suggest that CM, conversely to JM, were unable to discriminate visually between the different age classes. However, LT in CM subject showed different patterns from FL results. Figure 5 shows the LTs for the novel and familiar stimuli in the two order conditions. No significant main effect was found for novelty (F*_1,38_* = 2.56, *P* = 0.118) and order condition (F*_1,29_* = 0.116, *P* = 0.736); however, a significant interaction effect was found between the novelty category and order condition (*F_1,38_* = 10.67, P = 0.0023), indicating that the effect of novelty differed between the AI and IA conditions. The analysis of parameter coefficients in the GLMM revealed that the LT for the novel image was significantly longer than that for the familiar image in the AI condition, whereas no difference in LT was found in the IA condition (Figure 5, Table 2). This shows that in CM subjects, the LT for the novel stimulus was significantly longer than that for the familiar stimulus only when the novel stimulus was the infant image. These results are similar to those of the JMs, even though the stimuli depicted a completely unfamiliar species. The analysis of parameter coefficients in the GLMM also revealed that the LT for the novel image in AI condition (here, infant image) was significantly longer than those in the IA condition (adult), whereas LT for the familiar image in AI condition (adult) was significantly shorter than those in the AI condition (infant; see Figure 5, Table 2). This suggested that CM likely looked at infant images regardless of the stimulus category in the familiarization phase. The finding that CMs looked at infant images significantly longer than adult images suggests that CM subjects, like JMs, preferred to look at unfamiliar heterospecific JM infant images over JM adult images.

**Table 2:** GLMM parameter estimate coefficients in Experiment 2.

Estimated parameter contrasts	Average ± SE	Df	*t*-value	*P*
Difference between novel and familiar images in the AI condition	23.38±6.85	38	3.41	0.0015
Difference between novel and familiar images in the IA condition	9.11±7.21	38	1.26	0.21
Difference between AI and IA conditions for the familiar image	−17.94±7.03	29	2.55	0.0163
Difference between AI and IA conditions for the novel image	14.55±7.03	29	2.07	0.048

## Discussion

Our results are the first reported evidence of a spontaneous preference for infant images in nonhuman animals, and our study is a unique experimental demonstration in terms of monkey cognitive features of age class category.

On the one hand, in the VPC recognition tasks with Japanese macaques, JM subjects looked first at novel stimulus in both order conditions, showing a consistent novelty preference within a short-time scale. This indicates that the subjects could discriminate between paired stimuli of different age categories. Moreover, longer LTs provoked by the novelty preference in the test phase were found only in the AI order condition. Our VPC task using infant and adult image categories revealed a difference in LTs between the AI and IA conditions. When adult images were used in the familiar phase (AI condition), the monkeys looked at the novel infant image longer than the adult image. This finding suggests the monkeys possessed the ability to visually discriminate between categories and that they were able to show a preference: first by recognizing the infant image as novel, and second, by showing a preference for the image of the infant over that of the adult. In the IA condition, when the infant image was used in the familiar phase, the LTs for the adult and infant images were equal in the test phase. If the LTs were not influenced by image presentation in the familiar phase, they would have been longer for the infant images than for the adult images in the IA condition; however, the results did not show an asymmetric effect. In the IA condition, it is likely that the preference for the novel category (the adult image) was offset by the general preference for the infant image, resulting in equal LTs. Interestingly, the same asymmetric effect was found in VPC tests for gender discrimination using face stimuli in both human infants and Japanese macaques, concluding for a spontaneous preference to look at female faces [22], [23]. Thus, the results in the IA condition support the monkeys' spontaneous visual recognition and spontaneous preference abilities in JM subjects.

On the other hand, these results were only partially confirmed at the heterospecific level when testing Campbell's monkeys subjects as FL and LT patterns were respectively different and similar between JM and CM subjects. In the VPC tasks with CM subjects, the side of the first look in the test phase did not differ between novel and familiar stimuli, showing no novelty preference within a short-time scale. This indicates that CMs could not discriminate between paired stimuli of different age categories. Contrary to FL results, patterns of LTs in CMs were similar with those of JMs. The longer LTs provoked by the novelty preference in the test phase were found only in the AI order condition. Moreover, the subsequent analysis revealed that infant stimuli consistently attracted their gaze over adult ones. This suggests that heterospecific infant images were more attractive and preferred for CMs despite the absence of discriminative ability. Along with the JM results, our analysis suggested that the discriminative ability is species-specific, while the spontaneous preference for infant images is a universal cognitive feature that goes beyond species differences. Of course, further studies with more species are now needed to confirm the universality of this trait.

The results of our study present the possibility that nonhuman primates recognize age class categories, at least the infant class with species differences. For animals that live in social groups, such as nonhuman primates, recognition of age class would influence social interactions. One of the examples is the non-maternal care of an infant. In many primate species [12], [34], [35], [36], [37], [38], adults, most often females but sometimes males [39], [40], pay particular attention to caring for and interacting with young infants. It is possible that this allomaternal behavior is guided by physical features related to age class. Thus, the potential influence of recognition of age class on social interactions in nonhuman primates is supported by field observations, but has not been empirically tested. Of particular interest in our demonstration is the difference we observed between species. Our data suggested a species-specific process of age class recognition. Interestingly, similar species-specific processes have been found in face recognition in human infants and monkeys. The same VPC experiments in humans and monkeys have tested their recongnition of faces, and revealed their high sensitivity for own-race faces in humans or conspecific faces in monkeys, known as “own-race effects” (e.g. [14], [41]). It would be plausible that their ability of immediate age class recognition is specially tuned to conspecifics.

The debate about age concept in nonhuman primates remains open. Although our results strongly suggest the ability of age class recognition in JM subjects, we might not deny the alternative explanation by the model of saliency-based visual attention (for review, [42]). In this model, bottom-up process, like perceptions of roundness, brightness, contrast, proportion included in the stimulus, is more important than higher order cognitive process like as age class recognition. Recently, perceptual experiments in humans revealed that infant faces catch their visual attentions more quickly than adult faces [43], [44]. Attentional capture by infant faces might be caused by visual saliency included in infant faces (e.g., roundness of cheek and eyes), without any cognitive process of age class recognition. For the precise conclusion on age class concepts in monkeys, we need further experiments, using a direct way, e.g., conceptual visual discrimination tasks or matching-to-sample tasks based on operant conditioning.

Our results showing that JMs and CMs spent more time looking at images of infants than of adults contribute to evolutionary biology by providing evidence for a universal preference for infants in nonhuman primates, analogous to the human preference for baby schema proposed by Konrad Lorenz. He defined the baby schema as a set of infantile physical characteristics, such as a round face, large head, big eyes, high and protruding forehead, chubby cheeks, small nose and mouth, short and thick extremities, and plump body shapes. Lorenz hypothesized that these features were innately perceived as cute and motivated caretaking behavior in humans, acting as a “social releaser” [1]. Lorenz's theory held that the evolution of this adult perception or social cognition was shaped by the selective advantages of the survival of immature offspring. Several empirical psychological [26], [27], [28], [29], [30], [31], [44], [45], [46], [47], [48], [49], endocrinological [50], [51], and neuroimaging studies [43], [52] have supported his ideas. However, all such studies have been conducted in humans. As infantile physical features are present in other mammalian and avian species, it is surprising that no study has been conducted in nonhuman animals. Furthermore, an equivalent preference for babies is plausible in other animals, particularly in mammals and birds, because their infants are born immature and need adult nurturing. It is often argued that the nonhuman primate preferential interaction with infants is a social strategy. For example, young females use allomaternal care as “training” for maternal skills and/or to trade for grooming or protection [53], whereas males use infants as “buffers” during agonistic interactions [12], [34], [35], [36], [37], [38]. Conspecific and heterospecific adoption of infants has been observed in nonhuman primates [54]. The present study shows that beyond any social strategy, the attractiveness of infants may be an instinct in nonhuman primates and contribute to some extent to those aforementioned behaviors. Based on our results, we conclude that images of infants attract the visual attention of monkeys more than adult images and that this interest in infants transcends species differences. Our results suggest that paying special attention to infants is a universal and fundamental cognitive mechanism that evokes maternal nurturing in nonhuman primates. Moreover, these findings may indicate the evolutionary origins of the innate preference for baby schema in humans. To support an universal preference for infant images, we now need to extend our investigation to other primate and non-primate species.

## Materials and Methods

### Ethical note

All procedures in Experiments 1 and 2 complied with the Guide for the Care and Use of Laboratory Primates (Third Edition of Primate Research Institute, Kyoto University), approved by the Ethics Committee of the Primate Research Institute of Kyoto University (#2011-031).

### Subjects

Experiment 1 comprised 11 female Japanese macaques (JMs) ranging in age from 2 to 6 years old (two 2-year-olds, three 4-year-olds, five 5-year-olds, and one 6-year-old). They were all born in social groups and housed in open enclosures at the Primate Research Institute of Kyoto University (Japan), and lived with their own mother and group members. During our experiment, they were moved to individual cages, allowing them to visually and vocally interact with other monkeys.

Experiment 2 included 10 female Campbell's monkeys (CMs) ranging in age from 5 to 19 years old (one 19-year-old, two 18-year-olds, one 16-year-old, two 15-year-olds, one 7-year old, one 6-year-old, two 5-year-olds). They were all born in the same social group and housed in an open enclosure at Rennes 1 University, Station Biologique de Paimpont (France) and remained in their natal group. They were housed in an indoor (9.60 m × 1.65 m × 3.25 m) – outdoor (29 m × 9.80 m × 4.20 m) enclosure enriched with cords, branches, and litter (natural outside, straw inside). The CM group lived next to other guenon and mangabey species but were totally naive to the JM species. All monkeys in both species were fed daily with monkey pellets and fruits, and received water *ad libitum*.

### Apparatus in Experiment 1

The VPC tasks for the JM subjects were performed in a custom-made experimental box (450 mm W × 450 mm D × 600 mm H) in a sound-attenuating chamber (RE-246, Tracoustics Inc, Austin, TX, USA). Three sides of the experimental box were covered by transparent polycarbonate boards and the remaining side was the door of the cage. The monkeys were individually introduced into the experimental box through the openable stainless-steel board. A 22-inch LCD screen (ProLite E2208HDS, IiIyama, Japan) was placed on one side of the transparent polycarbonate board. The subject was thus allowed to look at the monitor through the transparent board. The LCD was connected to a computer placed outside of the sound chamber and the experimenter controlled stimulus presentation via a computer. The display resolution of the LCD was set to 1600×800 pixels. A small 1/3 inch pinhole on an infrared-sensitive charge-coupled device (CCD) camera (40 mm W × 36 mm H × 25 mm D, ITC-401, ITC, Japan) was placed at the center of the monitor and connected to a TV screen (LC-22K5, Sharp, Tokyo, Japan) outside of the sound chamber so the experimenter could monitor the gaze of the subjects. The CCD camera was simultaneously connected to a video camera (model, Victor, Japan) to record the subject's behavior during the experiment.

### Apparatus in Experiment 2

The VPC tasks for the CM were performed in an experimental room (1.5 m W × 2.5 H × 2.5 D) adjacent to the enclosure where the group lived. All monkeys had been trained to enter the room alone and sit on the small platform attached to the middle of the wired fence. For each trial, the experimenter isolated one subject from the enclosure in the experimental room. The room was made of brick (back), wood boards (sides), and wire fence (front). A 22-inch LCD monitor (DELL 2007FPb, USA) was placed outside of the experimental room so the subject could see it through the wire fence. The display resolution of the LCD was set to 1600×800 pixels, as in Experiment 1. The CCD camera was placed at the center of the monitor and connected to the video camera used to monitor and record the subject's behavior during the experiment. To make the setting equivalent to that of Experiment 1, the subjects were visually separated from the human experimenter by the placement of a black curtain between the monkey and the human monitor.

### Stimuli

JM images were used as the visual stimuli in both experiments. We prepared two stimulus categories, adult females (sexually mature, ≥4 years old) and infant females (<1 year old). None of the stimulus individuals were familiar to our JM and CM subjects. All stimulus individuals were shown from their front side with the whole body and the face clearly visible with a uniform black background, and with no emotional expression. The stimuli were made by cutting the whole body color image from the original photograph and reshaping it to fit the height of the body image to 300 pixels. Consequently, all image sizes were within an area of 300×300 square pixels. The average luminance and contrast were adjusted to equivalent values using Adobe Photoshop CS5. Six stimuli were prepared for each of the two stimulus categories.

### Procedure

A single VPC task trial consisted of four phases: the fixation, familiarization, re-fixation, and test phases (Figure 1). Prior to initiation of the trial, we displayed a fixation cross as a fixation point at the center of the monitor to draw each monkey's gaze/attention to that point. After confirming the subject's gaze direction, we initiated the VPC task. In the familiarization phase, we presented a single photo image at the center of the monitor for 5000 ms. Then the re-fixation phase began, in which the same fixation cross was displayed at the center of the monitor for 500 ms. The final test phase was then initiated. We simultaneously presented a pair of photo stimuli consisting of two new images, one belonging to the same stimulus category as the image in the familiarization phase (familiar stimulus) and the other one belonging to the other category (novel stimulus). The horizontal distance between the left and right image centers was set at 1300 pixels. The presentation time during the test phase was 5000 ms. The trial was performed once a day for each subject.

The tasks included two order conditions: adult-infant (AI) and infant-adult (IA). The adult image was the familiar stimulus and the infant image was the novel stimulus in the test phase of the AI condition, and vice versa for the IA condition. Two trials per subject were performed for each order condition, each using new pictures, counterbalancing the side-by-side position of the paired stimuli in the test phase. Consequently, four trials were performed for each subject. Experiment 1 was conducted in October and November, 2011, and Experiment 2 was conducted in December, 2011.

### Video analysis

We measured the side of the first look (FL) in the test phase, and the total looking time (LT) on each side during the paired stimuli presentation period of the test phase. To measure the FL and LT, coders blind to the conditions examined the video clips taken during the test phase and coded the subjects' gaze directions into three categories: looking to the left side, looking to the right side, and not looking at the screen. The blind codes were assigned with an accuracy of 33 ms per video frame using a custom-made program. After coding, the FLs and LTs were assigned to two stimulus categories.

### Statistical analysis

To examine which of novel or familiar category was first looked in the test phase, we conducted a general linear mixed model (GLMM) procedure to estimate fitted models using the glmmML function contained in the glmmML package of the R statistical environment for statistical computing (ver. 2.14.1; R Development Core Team). In the model, the ordered factor (AI, IA) was explanatory fixed factor, and subject was a random factor. For the model fitting, we binomially scored cases when FL side was novel as 1 and cases when FL was familiar as 0. A binomial distribution was used with a logit link function. In the model, we examined a statistical significance of the parameter coefficients estimated. In the logit transform, intercept of GLMM with binominal distribution represents ln (*p*/*q*), where *p* and *q* stand for probability of FL for novel side and for familiar side. If *p* equals *q*, intercept would be estimated around 0. Otherwise, intercept would be statistically differed from 0.

To examine the effects of the order conditions (AI, IA) and stimulus novelty category in the test phase (novel versus familiar) on total LTs, we conducted a GLMM using the lme function in the nlm package of the R. In the model, the ordered factors (AI, IA) and the novelty factors (novel, familiar) were explanatory fixed factors, and trials nested within subjects were the random factors. The analysis of variance (ANOVA) function in the R software was used to determine statistical significance. If an interaction effect was found, we conducted further analysis of the parameter coefficients in the model. The statistical analyses were performed separately for Experiment 1 and 2. P-values <0.05 were deemed to be statistically significant.
